# Assessment of protein silver nanoparticles toxicity against pathogenic *Alternaria solani*

**DOI:** 10.1007/s13205-016-0515-6

**Published:** 2016-09-21

**Authors:** Sobhy I. I. Abdel-Hafez, Nivien A. Nafady, Ismail R. Abdel-Rahim, Abeer M. Shaltout, José-Antonio Daròs, Mohamed A. Mohamed

**Affiliations:** 1 Botany and Microbiology Department, Faculty of Science, Assiut University, Assiut, 71516 Egypt; 2 Plant Pathology Research Institute, Agricultural Research Center, Giza, 12655 Egypt; 3 Instituto de Biología Molecular y Celular de Plantas (Consejo Superior de Investigaciones Científicas-Universidad Politécnica de Valencia), Avenida de los Naranjos, 46022 Valencia, Spain

**Keywords:** Silver nanoparticles, Mycosynthesis, Pathogen, *Alternaria solani*, Antifungal activity

## Abstract

Mycogenic synthesis of silver nanoparticles (AgNPs) was carried out in the present investigation using an aqueous extract of endophytic non-pathogenic *Alternaria solani* F10 (KT721914). The mycosynthesized AgNPs were characterized by means of spectroscopic and microscopic techniques. The surface plasmon resonance found at 430 nm confirmed the formation of stable AgNPs for several weeks at room temperature. Also, the results revealed the formation of spherical and monodispersed AgNPs with an average size of 14.8 ± 1.2 nm. The FT-IR spectrum suggested that the fungal extracellular proteins and secondary metabolites had the role in Ag reduction and AgNPs capping of which protein Ag nanoconjugates were formed. Furthermore, the mycosynthesized AgNPs exhibited potent antifungal activity against different pathogenic isolates of the same *Alternaria solani* fungus, the causal pathogen of tomato early blight disease. The antifungal efficiency of the AgNPs at 1, 5 and 10 ppm were evaluated for 8 days after incubation by measuring the inhibition rate of fungal radial growth. The results were further supported by investigating fungal hyphae morphology alteration by scanning and transmission electron microscopy. Treated fungal hyphae showed formation of pits and pores. Also, the mycosynthesized AgNPs were able to pass and distribute throughout the fungal cell area and interact with the cell components.

## Introduction

Nano-biotechnology has emerged as one of the fastest growing areas of research in modern materials science and technology (Thakkar et al. 2010). Synthesis of nanoparticles has generated much interest in academia, as well as industry, because it bridges the gap between bulk materials and atomic molecular structures (Moradi et al. 2013). Owing to their unique physical and chemical properties, silver nanoparticles (AgNPs) are increasingly being applied in a variety of fields, including optics, electronics, mechanics, catalysis, energy science, medicine and agricultural technology (Ensafi and Karimi-Maleh 2010; Elyasi et al. 2013; Sadeghi et al. 2013). AgNPs are known to exhibit a broad spectrum of biocidal activity towards many bacteria, fungi and viruses (Zachariadis et al. 2004; Kumar and Sujitha 2014; Abd-Alla et al. 2016; Netala et al. 2016). From a therapeutic perspective, AgNPs are finding their way as antimicrobial (Kim et al. 2009a), anti-inflammatory (Nadworny et al. 2008), anti-angiogenic (Gurunathan et al. 2009) and antiviral (Rogers et al. 2008) agents. Although chemical and physical methods have allowed successful production of well-defined AgNPs, they are usually costly and involve the use of toxic reducing and capping reagents (Qin et al. 2010). In contrast, biological synthesis of AgNPs is a promising strategy that relies on natural products obtained from fungi, bacteria, algae, viruses, or plants (Narayanan and Sakthivel 2010; Das et al. 2014; Kagithoju et al. 2015; Netala et al. 2016). Fungi are one of the best options to produce AgNPs, due to the vast repertoire of proteins, enzymes, and other bioactive secondary metabolites that they produce and possess redox activity (Birla et al. 2009; Metuku et al. 2014).

More specifically, fungal endophytes have been recognized as important sources of a wide variety of structurally unique, bioactive natural products, which offer an enormous potential in medical, agricultural and industrial uses (Zhang et al. 2007). However, up to date, very few studies have investigated the biological synthesis of AgNPs using fungal endophytes (Tanvir et al. 2012; Liu et al. 2013).

*Alternaria solani* (Ellis & Martin) Jones & Grout is a soil inhabiting, air-borne fungal pathogen responsible of tomato leaf blight, and collar and fruit rot (Datar and Mayee 1981). It causes reduction in crop quantity and quality. Current strategies to control early blight disease consist of preventing wetness on leaf surface during long periods of time, development of host plant resistance and application of fungicides. Synthetic chemical fungicides are widely used in conventional agriculture to control plant diseases. Different chemical fungicides including Ridomil gold plus, Mancozeb, copper oxychloride, carbendazim, captafol and benomyl have been used today to control tomato early blight caused by *A. solani* (Chohan et al. 2015; Saharan et al. 2015). However, environmental toxic hazards caused by excessive use of those fungicides pose health problems as modern society is becoming more conscious (Kim et al. 2009b). In addition, pathogens can derive resistance against fungicides (Namanda et al. 2004; Kirk et al. 2005). Also, pathogen populations that develop resistance to one fungicide may automatically and simultaneously become resistant to other related fungicides. Therefore, scientists in the agricultural field are searching for alternative eco-friendly and less capital intensive approaches to control plant diseases and crop protection. Nanoparticles may act upon pathogens in a way similar to chemical pesticides. Nanomaterials can also be used as carriers of active ingredients of pesticides to the target pathogens (Khan and Rizvi 2014). Biologically synthesized AgNPs in different formulations and with different shapes and sizes should rely on products for controlling various plant fungal pathogens in a relatively safer way compared to synthetic fungicides.

In the present study, we report the production of AgNPs using extracts from an endophytic non-pathogenic isolate of *Alternaria solani*. The mycosynthesis process was carried out under ambient conditions, without the addition of any external chemical reducing agent. We also report the antifungal activity of the mycosynthesized AgNPs compared to the chemical fungicide (Ridomil gold plus) against different pathogenic isolates of the same fungus *Alternaria solani*. Finally, we also study the possible action mechanisms of the mycosynthesized AgNPs.

## Materials and methods

### Sample collection and isolation of endophytic fungi

Fresh healthy leaves of tomato (*Solanum lycopersicum* L.) plants were collected from Aswan Governorate, Egypt, to isolate endophytic fungi. The collected samples were packed directly into sterilized polyethylene bags and transferred to the mycological laboratory, Botany and Microbiology Department, Assiut University. Leaves were thoroughly washed with running tap water and aseptically cut into small segments (5 × 5 mm). All segments were rinsed with distilled water and surface sterilized following the sequence: 70 % ethyl alcohol for one minute, and then transferred to a solution of 2.5 % sodium hypochlorite for 3.5 min, followed by a treatment with 70 % ethanol for 30 s. The segments were finally placed on Petri dishes containing potato dextrose agar (PDA) medium containing 250 mg L^−1^ streptomycin. The plates were incubated at 26 ± 2 °C for a period of 8 days and observed at regular intervals for fungal growth. The hyphal tips of the endophytic fungi growing out from the plant tissues were cut with a sterile Pasteur pipette and transferred onto new PDA plates for better isolation. After incubation at 26 ± 2 °C for 8 days, fungal endophyte identification was performed according to morphological characteristics. Percent colonization frequency (% CF) of endophytic fungi was calculated according to Petrini and Fisher 1988: colonization frequency (%) = (total number of segments colonized/total number of segments) × 100.

### Screening of endophytic fungi for mycogenic synthesis of AgNPs

All the isolated endophytic fungi were screened for the mycogenic synthesis of AgNPs. Isolated endophytic fungi were grown aerobically in potato dextrose broth containing infusion of 250 g potato and 20 g dextrose per liter of distilled water. The inoculated flasks were incubated in an orbital shaker at 26 ± 2 °C and agitated at 120 rpm for 3 days. After incubation, the profusely grown fungal mat was washed extensively using sterile double distilled water to remove any medium component. In general, 10 g (wet weight) of fungal mat was added to 100 mL sterile double distilled water in an Erlenmeyer flask and agitated again at 120 rpm for 48 h at 26 ± 2 °C. Then, the cell filtrate was obtained by filtering through Whatman filter paper no. 1. The filtrates were mixed with 100 mL of 1 mM silver nitrate (Sigma Aldrich) in Erlenmeyer flasks and the mix incubated at room temperature (~22 °C) in the dark. Cell-free filtrates without silver nitrate solution and silver nitrate solution with no cell filtrate added were run as controls under similar experimental conditions. Color change was observed for up to 48 h (Fayaz et al. 2010).

### Molecular identification of *Alternaria solani* isolates

Among the isolated endophytic fungi tested, the non-pathogenic *Alternaria solani* isolate was further chosen for molecular characterization. The three pathogenic *Alternaria solani* isolates were also molecularly identified. The genomic DNA was isolated by grinding about 100 mg of frozen fungal mycelia with liquid nitrogen using a mortar and pestle and mixed with 1 mL of 4 M guanidinium thiocyanate, 0.1 M sodium acetate pH 5.5, 10 mM ethylenediaminetetra acetic acid (EDTA), 0.1 M 2-mercaptoethanol. Extract was clarified by centrifugation and the supernatant loaded into a silica gel spin column (Wizard Plus SV Minipreps DNA Purification, Promega, USA). Column was washed with 70 % ethanol, 10 mM sodium acetate pH 5.5 and DNA eluted with 50 µl of 20 mM Tris–HCl, pH 8.5.

### Ribosomal DNA amplification and sequencing

Ribosomal internal transcribed sequence (ITS) was amplified by PCR using primer pairs ITS1 (5′-CTTGGTCATTTAGAGGAAGTAA-3′) and ITS4 (5′-TCCTCCGCTTATTGATATGC-3′) (Gardes and Bruns 1993). Fungal DNA (1 µL) was amplified in a 20-µL reaction with 0.4 U Phusion DNA polymerase (Thermo Scientific) in the presence of 0.2 mM dNTPs, 3 % dimethyl sulfoxide, 0.5 µM each primer and HF buffer (Thermo Scientific). Reaction consisted of an initial denaturation for 30 s at 98 °C, followed by 30 cycles of 10 s at 98 °C, 30 s at 55 °C and 30 s at 72 °C, and a final extension of 10 min at 72 °C. PCR products were separated by electrophoresis in a 1 % agarose gel run for 75 min in buffer TAE (40 mM Tris, 20 mM sodium acetate, 1 mM EDTA, pH 7.2) and visualized using a UV transilluminator after ethidium bromide staining. The PCR product corresponding to ribosomal ITS, according to electrophoretic migration, was eluted from the gel using silica spin columns (DNA Clean and Concentrator, Zymo Research). Both strands of amplified ribosomal ITS DNA were sequenced using primers ITS1 and ITS4. The consensus sequence was used to search for homologous sequences using the BLAST search program at National Center for Biotechnology Information (NCBI; http://www.ncbi.nlm.nih.gov).

### Characterization of mycosynthesized AgNPs

The production of AgNPs using an extract of non-pathogenic endophytic *Alternaria solani* was preliminarily confirmed by visual observation of solution color change into pale brown. The morphological, structural and chemical compositions of mycosynthesized AgNPs were further analyzed.

Dialysis experiments were carried out to confirm the purity of mycosynthesized AgNPs and the absence of free Ag + ion. The dialysis bags were filled with 4 mL AgNPs dispersion and immersed in 296 ml bi-distilled water (Loza et al. 2014). The dialysis was carried out under slow stirring with a magnetic stirrer at room temperature.

### UV–vis spectroscopic analysis

The mycosynthesized AgNPs were characterized by UV–vis spectroscopy periodically for 1 month to observe the formation of stable AgNPs by the action of the fungal extract. Small aliquots (2 mL) of the colored suspended particles were loaded in a quartz cuvette and analyzed through a wavelength scanning ranging between 300 and 800 nm at different time intervals with distilled water as a reference. A PerkinElmer Lambda 950 UV/Vis spectrometer was used.

### High resolution transmission electron microscopy (HR-TEM)

The colored mixture of AgNPs was centrifuged at 14,000 rpm for 15 min. The supernatant was discarded and the nanomaterial pellets were dispersed with deionized water and centrifuged three times to remove the free entities and unbound biological molecules from the formed AgNPs. The purified sediments were dried at 60 °C for further characterization.

For HR-TEM measurements, the sample solution was pipetted onto a carbon-coated copper grid (carbon type-B, 300 mesh, Ted Pella, Inc., Redding, CA, USA). The sample-loaded grid was air dried under vacuum for 3 h. The TEM micrograph images were recorded using a JEOL 1200 EX instrument operated at an accelerating suitable voltage (kV). The hydrodynamic diameter and the zeta potential of the AgNPs were measured by dynamic light scattering (DLS) using a Malvern Zetasizer Nano ZS 90 (Worcestershire, UK). Additionally, selected area electron diffraction (SAED) of the nanoparticles was also analyzed.

### X-ray diffraction (XRD) and energy-dispersive spectroscopy (EDS) analysis

The XRD patterns were collected on a Bruker AXS D8 Advance X-ray diffractometer with Cu K*α* radiation of wavelength 1.541 Å and scanning angle 2*θ* in the range of 10°–80°. EDS analysis was performed for identifying the elemental composition of the biosynthesized AgNPs by EDS using an INCA Energy TEM 200 with analysis software (JEOL).

### Fourier transform infrared (FT-IR) analysis

FT-IR analysis was performed to determine the possible functional groups in the fungal extract responsible for bio-reduction of Ag^+^ ions and formation of AgNPs. The FT-IR measurement was carried out for 10 mg of AgNPs powder mixed with a pinch of potassium bromide (Himedia FT-IR graded) in a crucible. The mixure was made into pellet by hydraulic press and the pellet was then analyzed in Jasco FT/IR-6300 equipped with JASCO IRT-7000 Intron Infrared Microscope using transmittance mode operating at a resolution of 4 cm^−1^ (JASCO, Tokyo, Japan) (Siddique et al. 2013).

### Antifungal activity of AgNPs

The antifungal activity of AgNPs was measured on three pathogenic isolates of *Alternaria solani* by the agar dilution method. The agar medium was supplemented with three concentrations of biogenic AgNPs (1, 5, 10 ppm). A disc (1.5 cm) of mycelial growth of the phytopathogenic fungus *Alternaria solani*, taken from the edge of 8 day old fungal culture, was placed in the center of each plate. The inoculated plates were then incubated at 25 °C for 8 days. The effects of AgNPs treatments were evaluated by measuring the radial growth of fungal colonies (Kim et al. 2012):

$${\text{Inhibition}}\,{\text{rate}}\,(\% ) = R - {r \mathord{\left/ {\vphantom {r R}} \right. \kern-\nulldelimiterspace} R} \times 100$$

“Where* R* is the radial growth of fungal hyphae on the control plate and* r* is the radial growth of fungal hyphae on the plate supplemented with AgNPs”. The commercial chemical fungicide (Ridomil gold Plus at 2 gL^−1^) was used as a positive control. All experiments were conducted in triplicate under sterile conditions.

### Effect of AgNPs on pathogenic *Alternaria solani* mycelial morphology

The mycelia of *Alternaria solani* F11 (KT721909) were treated with 0.5 of 1 mM of AgNPs aqueous solution and incubated under ambient conditions. The morphological changes in the treated fungal tissue were examined using a field emission scanning electron microscope (FE-SEM; S-4700, Hitachi, Japan) at an accelerating voltage of 5.0 kV.

Ultra-thin sections of treated *A. solani* mycelia were prepared using an ultra-microtome (Leica Ultracut R) instrument. The exposed hyphae sections were then fixed within 2 % glutaraldehyde in 0.05 M sodium phosphate for 2 h and washed with 0.05 M sodium phosphate. Subsequently, they were subjected to dehydration with serial concentrations of ethanol for 5–10 min at each concentration followed by two rinses in absolute ethanol for 15 min for dryness. Dehydrated samples were infiltrated in Spurr’s resin at the following proportions of resin/ethanol: 15:85, 30:70, 60:40, 90:10, and finally filtrated and kept overnight in 100 % of the resin. The prepared thin sections were thin fixed on carbon-coated copper grids and examined by a Hitachi H-7500 HR-TEM microscope at 100 kV to investigate the interaction of AgNPs with the fungal pathogen cell components. Uranyl acetate (UA) and lead citrate (LC) were used as staining solutions and rinsing was carried out with distilled water.

### Effect of AgNPs on pathogenic *Alternaria solani* nucleic acid

DNA damage study was performed according to the protocol of Vahdati and Sadeghi (2013). DNA amplified by PCR was treated with mycosynthesized AgNPs (1 mM) and incubated for 1 h. DNA without nanoparticle treatment served as a control. After treatment DNA was separated by electrophoresis using a 1 % agarose gel at 75 V for 30 min. The gel was stained with ethidium bromide and then subjected to UV irradiation to visualize the DNA bands.

## Results and discussion

### Identification of endophytic fungi in tomato leaves

From the 40 analyzed leaf fragments of tomato plants, eight fungal species belonging to seven genera were isolated (Table 1). Fungal species were identified according to their morphological characters. Table 1 also includes their frequencies of colonization. The most predominant genus was *Alternaria*, which showed 65 % colonization frequency. Meanwhile, *Alternaria alternata* was the most prevalent species with a frequency of 57.5 %. The incidence of *Alternaria solani* as an endophytic fungus in the samples tested was 7.5 %. This is an interesting result because some strains of *Alternaria soloni* cause an important disease in tomato crops. All isolated endophytic fungi were screened for the mycogenic synthesis of AgNPs. *Epicoccum nigrum*, *Curvularia lunata*, *Alternaria solani*, *Fusarium oxysporum* and *Penicillium* sp. allowed efficient synthesis of AgNPs. However, based on preliminary pathogenicity tests, particle stability and rate of synthesis, *Alternaria solani* was chosen to proceed with our research.

**Table 1:** The composition of the isolated endophytic fungal taxa and frequency of colonization (%) per 40 segments

Endophytic fungi	No. of records	Frequency (%)
*Alternaria alternata* (Fr.) Keissl	23	57.5
*Alternaria solani* (Ellis & Martin) Jones & Grout	3	7.5
*Aspergillus terreus* Thom	9	22.5
*Cladosporium oxysporum* (Berk. & Curtis)	13	32.5
*Curvularia lunata* (Wakker) Boedijn	12	30
*Epicoccum nigrum* Link	5	12.5
*Fusarium oxysporum* Schltdl	8	20
*Penicillium* sp.	7	17.5

Accurate identification of both the endophytic non-pathogenic isolate and three pathogenic variants of *Alternaria solani* were confirmed by sequence analysis of the nuclear ribosomal ITS region. The sequence of a 623 base pair DNA fragment corresponding to the ITS1-5.8S-ITS2 region supported the identification of the non-pathogenic isolate as *Alternaria solani* F10 (KT721914), showing 100 % identity with the sequence of the non-pathogenic endophytic *Alternaria solani* IA300 (AY154716). The same for the three pathogenic *Alternaria solani* isolates. A partial 18S rRNA gene sequence of approximately 624 base pairs of *Alternaria solani* F11 (KT721909), *Alternaria solani* F12 (KT721910) and *Alternaria solani* F14 (KT721911) had a sequence with 100 % identity to that of pathogenic *Alternaria solani* KT6 (F02664) available in the GenBank databases.

### Mycogenic synthesis of AgNPs

**Fig. 1:**
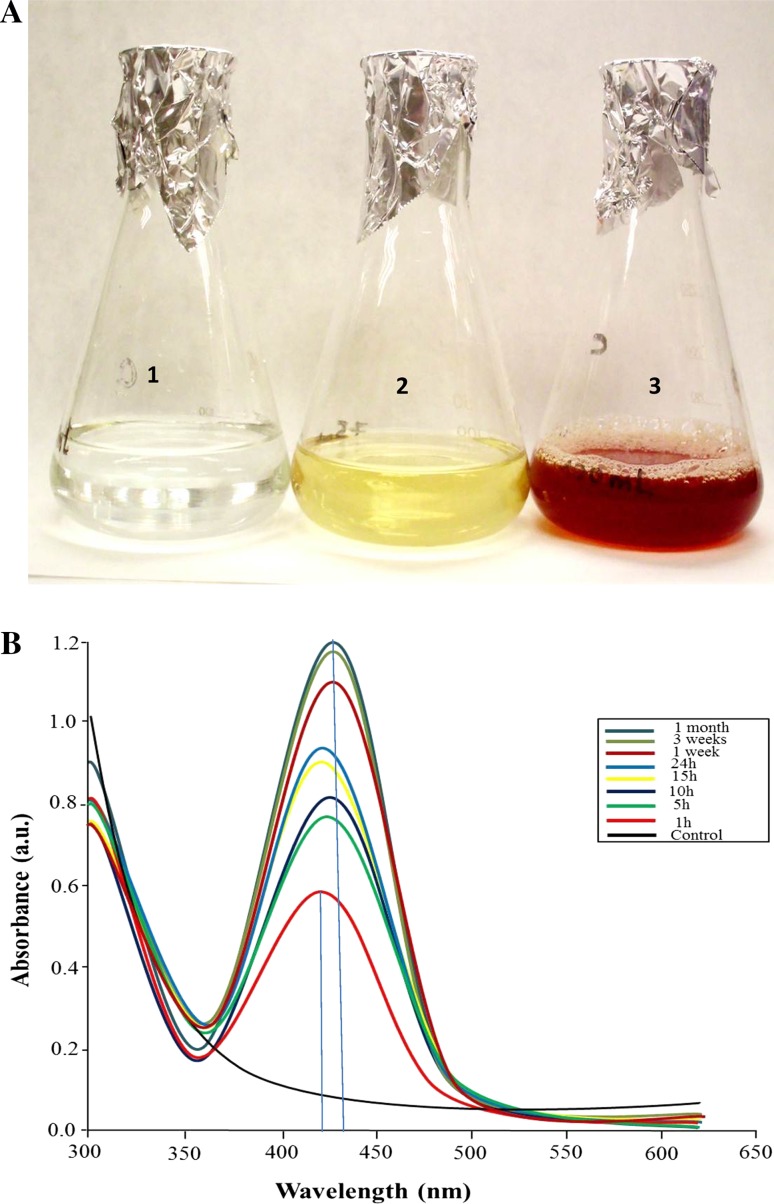
Mycosynthesis of AgNPs. **a** Formation of AgNPs. Erlenmeyer flasks containing (*1*) silver nitrate solution, (*2*) fungal extract, and (*3*) myconsynthesized AgNPs. **b** UV–vis spectra of the mycosynthesized AgNPs at different times, as indicated

The mycogenic synthesis of AgNPs using a fungal extract of the non-pathogenic isolate *Alternaria solani* F10 (KT721914) was confirmed by observing the color change of AgNO_3_ solution after challenging with the fungal extract. The mixture of AgNO_3_ and fungal extract changed rapidly after 5 h to a brown suspension, whereas AgNO_3_ without fungal extract showed no color change (Fig. 1a). This suggested that AgNPs can be synthesized using this particular fungal extract. Aqueous extracts of most fungi contain proteins, alkaloids, tannins, steroids, phenols, saponins, and flavonoids, which could induce the formation of nanoparticles by serving as reducing agents (Mohamed 2015; Abdel-Hafez et al. 2016). Moreover, the UV–vis absorption spectra showed a strong absorption peak centered at 430 nm which is characteristic for surface plasmon resonance of Ag (Azizi et al. 2013) and hence indicates the formation of AgNPs (Fig. 1b). The stability of the synthesized AgNPs was measured after storage for several weeks. The absorption peaks of the mycosynthesized AgNPs only slightly shifted from 420 to 430 nm, without a significant change in the spectral shape or the intensity (Fig. 1b). This result suggests that AgNPs are stable over a long period of time.

### Characterization of AgNPs

**Fig. 2:**
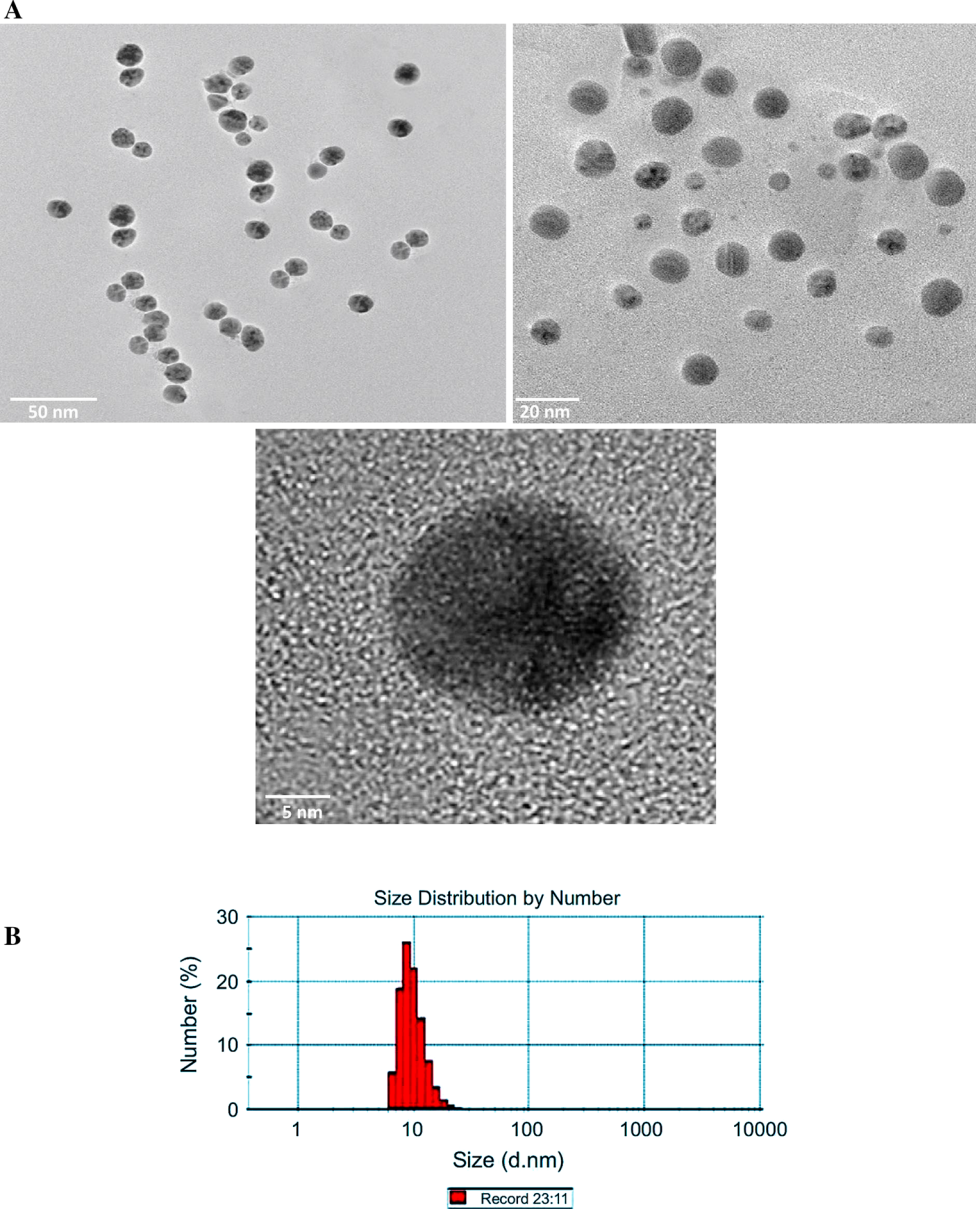
**a** HR-TEM images of the mycosynthesized AgNPs at different scales (50, 20 and 5 nm), as indicated. **b** Particle size distribution analysis

**Fig. 3:**
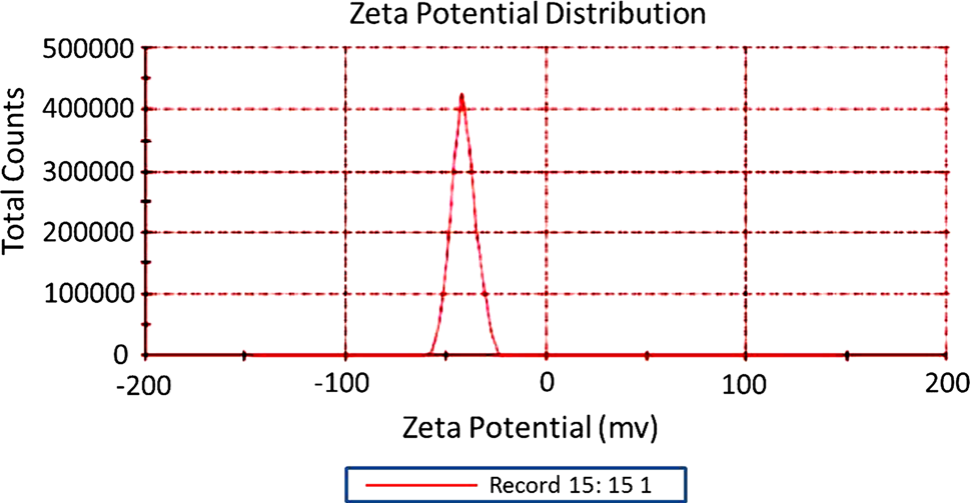
Zeta potential measurements of the mycosynthesized AgNPs

The size and morphology of the biosynthesized AgNPs were examined using HR-TEM. The results showed the formation of spherical and well dispersed nanoparticles with an average size distribution of 14.8 ± 1.2 nm (Fig. 2). A negative zeta potential of about −41.6 ± 0.5 mV was also observed in the current study that indicates an ideal surface charge of the formed AgNPs (Fig. 3). Moreover, the high absolute value of zeta potential revealed a high electrical charge on the AgNPs surface (Fig. 3), which can cause a strong repulsive force among the particles to prevent agglomeration and hence might be responsible for their high stability.

**Fig. 4:**
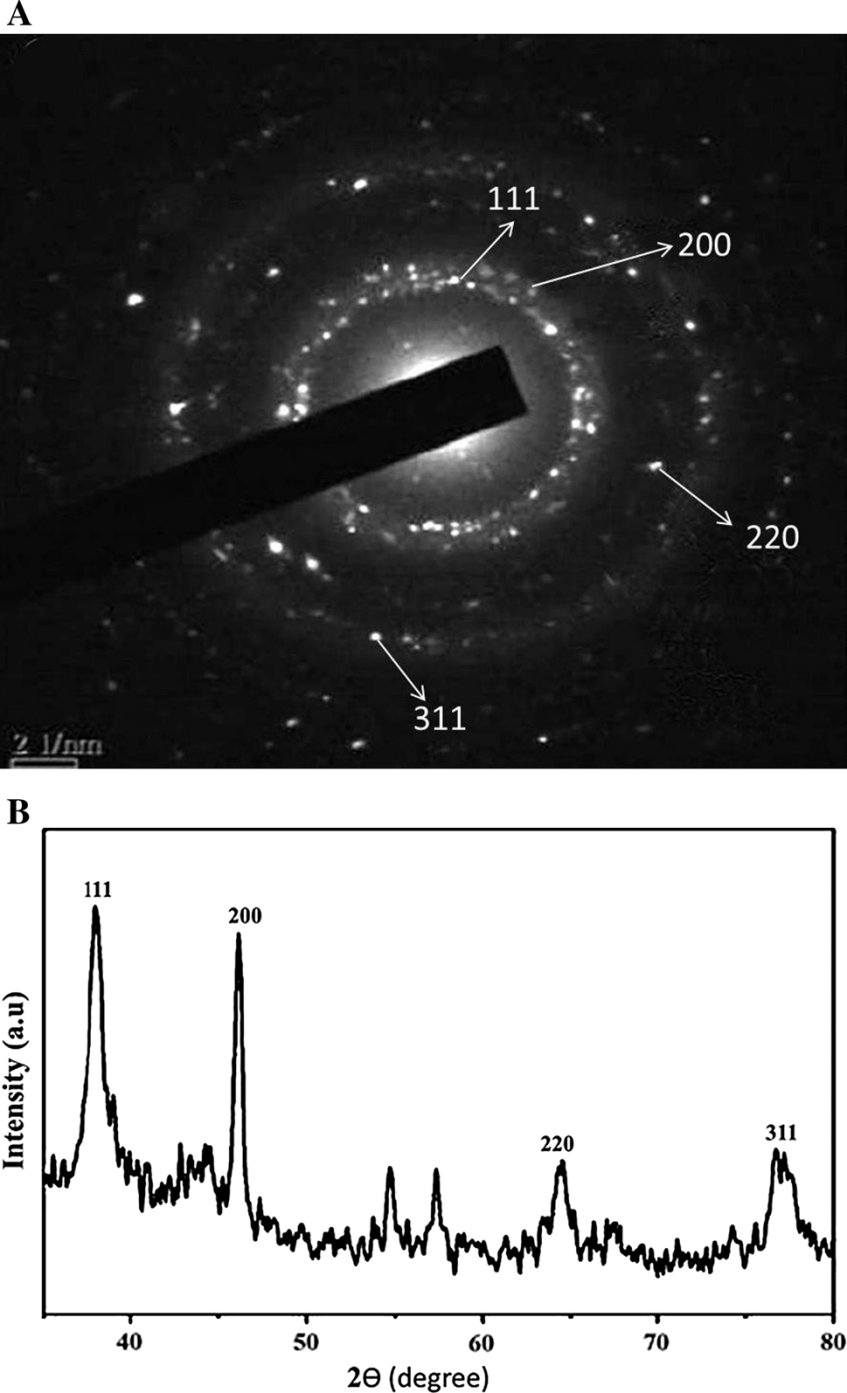
Characterization of AgNPs. **a** Selected area of electron diffraction (SAED) pattern of randomly selected AgNPs. **b** X-ray diffraction patterns of AgNPs

**Fig. 5:**
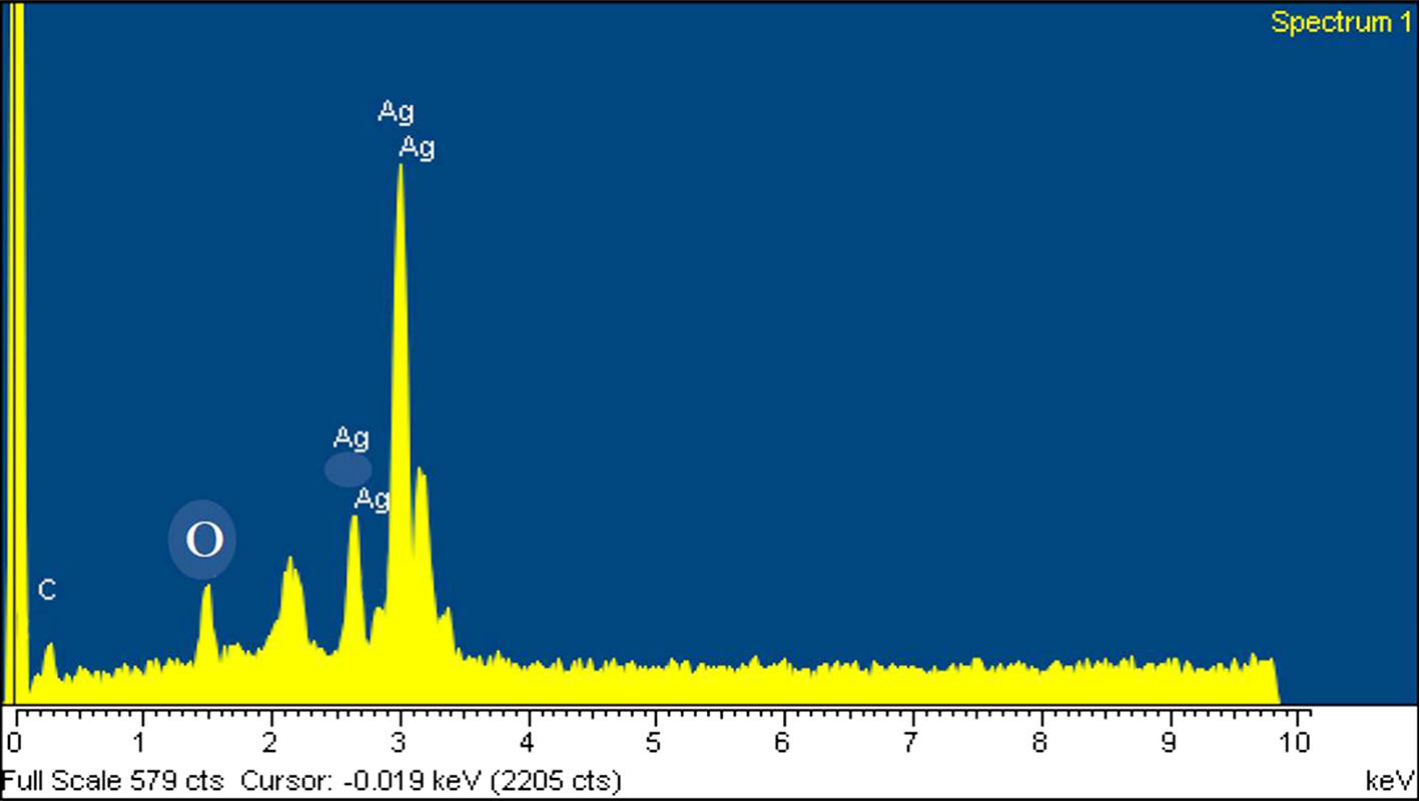
Characterization of AgNPs by EDX analysis displaying the purity and chemical composition of the mycosynthesized AgNPs

The spacing between clear lattice fringes in the HR-TEM image was found to be 0.23 nm that matches the (111) plane Ag crystal lattice (JCPDS 04-0783) (Fig. 4a). The SAED pattern shows circular rings, which can be indexed to the reflections from the (111), (200), (220) and (311) planes (JCPDS 04-0783) (Fig. 4a). These planes correspond to face-centered cubic (fcc) Ag and reveal the highly polycrystalline nature of the synthesized AgNPs. The more intense circular ring, which is closer to the center is due to (111) reflections. The second ring is indexed to the (200) reflections. The third and fourth ring belongs to (220) and (311) reflections, respectively (Fig. 4a). The crystalline nature of AgNPs was further confirmed by XRD analysis. The XRD pattern revealed the typical fcc structure of AgNPs. The XRD spectra showed four main characteristic Bragg diffraction peaks at 2θ values of nearly 36°, 46°, 65°, and 77° which correspond to (111), (200), (220), and (311) planes, respectively, of fcc silver nanoparticles (Fig. 4b). The diffraction peaks were consistent with standard database files (JCPDS card No. 04-0783), indicating that the synthesized nanoparticles were of pure crystalline in nature. This is in accordance with the SAED results. Moreover, EDS spectroscopy results confirmed the significant presence of pure metallic Ag with no other elemental contaminants (Fig. 5). The EDS spectrum confirmed the formation of AgNPs and showed a strong and typical optical absorption peak at approximately 3 keV, which was attributed to the SPR of the metallic Ag nano crystals (Azizi et al. 2013).

**Fig. 6:**
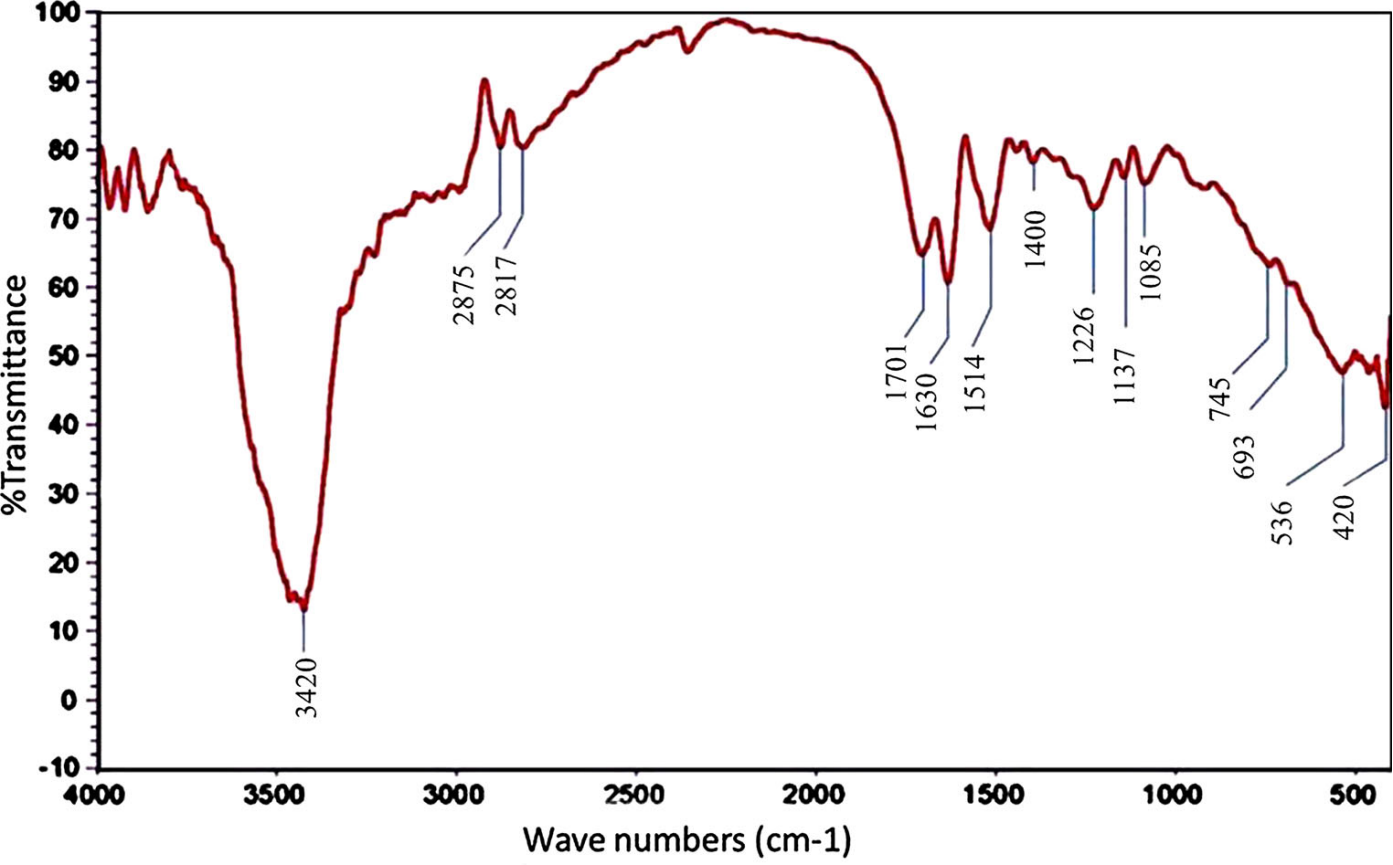
Characterizations of AgNPs by FT-IR spectroscopy displaying the function groups present in the mycosynthesized AgNPs

The FT-IR spectrum helped to identify specific functional groups present in the synthesized AgNPs, which may play the roles of capping and reducing agents. As shown in (Fig. 6), the IR spectrum displays intense bands at 3420, 2875, 2817, 1701, 1630, 1514, 1400, 1226, 1137, 1085, 745, 693, 536, and 420 cm^−1^. The broad band at 3420 cm^−1^ corresponds to the strong stretching vibrations of hydroxyl (–OH) group of phenolic compounds present in the fungal extract. The intense peaks at 1701 cm^−1^ can be attributed to the –C=O– and – C=C– stretching vibrations, which indicates the presence of flavonoids and terpenoids in the fungal extract of *Alternaria solani*. The medium absorption peak located at 1630 cm^−1^ can be identified as the amide group. This amide band occurs due to carbonyl stretch and N–H deformation vibrations in the amide linkage of proteins. It is well known that the metallic ions can bind to carboxylic groups, and therefore, this reveals opens the possibility of AgNPs bound to proteins through the carboxyl groups. The band at 1400 cm^−1^ corresponds to the C = N stretching vibration of aromatic amines. Thus, taking all together, these observations support that the AgNPs are surrounded by some proteins and secondary metabolites containing amine, alcohol, ketone and carboxylic acid functional groups.

Various mechanisms have been proposed in the literature for the formation of AgNPs from fungal extracts. Malik et al. (2014) stated that the metallic salts possess high reduction potential and their metals have the tendency for electron donation. This unique characteristic feature results in detachment of metallic ion (M^+^) from their native salt and reduction to the stable (M^0^) form with the help of fungal metabolites. In the present study, we would like to propose two mechanisms for the formation of AgNPs from *Alternaria solani* extract. The first is the oxidation of phenolic and hydroxyl groups present in the extract when challenged with AgNO_3_ solution. Then, the interaction between these phenolic groups and Ag ions would form intermediate complexes which will undergo oxidation and thus reduction of Ag^+^ to form AgNPs. The second mechanism is the metal chelating effect of biomolecules present in the fungal extract as proposed by Khan et al. (2013). Therefore, the Ag^+^ would bind with the biomolecules to form protein Ag nanoconjugation (Ag^0^). The stability is rendered by the proteins and the functional groups, particularly carboxylic and hydroxylic groups, and cysteine residues present in the extract.

### Antifungal activity of mycosynthesized AgNPs

The inhibitory effect of mycosynthesized AgNPs at different concentrations (1, 5 and 10 ppm) compared to the chemical fungicide (Ridomil gold plus 2gL^−1^) was analyzed in vitro. The results clearly showed that the AgNPs markedly exhibited higher antifungal activity. The mycelial growth of pathogenic *Alternaria solani* F11 (KT721909), *Alternaria solani* F12 (KT721910) and *Alternaria solani* F14 (KT721911) was inhibited to various extents by AgNPs (Table 2). A concentration of 10 ppm induced the highest levels of inhibition rate (%) of the three strains of pathogenic *A. solani* mycelial growth with values (88.9 ± 1.2, 87.8 ± 1.1 and 88.5 ± 1.0), respectively, compared to the chemical fungicide which showed values (61.4 ± 1.2, 60.9 ± 0.5 and 62.7 ± 1.3), respectively, after incubation period 8 days (Table 2). These results revealed that AgNPs are promising economic and safe antifungal agents against the phytopathogenic fungus at low concentrations of effective nanomaterials, even lower than the recommended concentration of the chemical fungicide, thus considered suitable for commercial use. Recently, the use of AgNPs as antimicrobial agents has become more common as technological advances make their production more safe and economical. One of the potential applications in which AgNPs can be utilized is in plant disease management as alternatives to the chemical fungicides which used in grams per liter (ex. Ridomil gold plus 2gL^−1^). This can contribute to minimize the risks and hazards of toxic chemical fungicides, especially on vegetables which produced for fresh consumption. Since several studies revealed that AgNPs display multiple modes of inhibitory action to microorganisms (Satyavani et al. 2011; Netala et al. 2016), they may be used for controlling various plant pathogens in a relatively safe way compared to synthetic fungicides (Stoimenov et al. 2002).

**Table 2:** Mean of inhibitory growth rate (%) of pathogenic *Alternaria solani* by the mycosynthesized AgNPs

Pathogen	Treatments	Concentration	Inhibition rate (%)		
			4 days	6 days	8 days
*A. solani* F11 (KT721909)	AgNPs	1 mg/L	43.6 ± 0.5	57.6 ± 0.7	57.8 ± 0.5
	Ridomil gold plus	5 mg/L	72.7 ± 0.4	80.0 ± 0.5	72.2 ± 0.5
		10 mg/L	96.4 ± 0.5	94.1 ± 0.5	88.9 ± 1.2
		2 g/L	66.3 ± 0.2	68.2 ± 0.5	69.7 ± 1.2
*A. solani* F12 (KT721910)	AgNPs	1 mg/L	28 ± 0.5	57.5 ± 0.5	55.6 ± 0.4
		5 mg/L	72 ± 0.5	78.75 ± 0.1	72.2 ± 0.5
		10 mg/L	94 ± 1.0	95 ± 0.2	87.8 ± 1.1
	Ridomil gold plus	2 g/L	64.3 ± 0.6	69.2 ± 0.3	68.9 ± 0.5
*A. solani* F14 (KT721911)	AgNPs	1 mg/L	45.5 ± 0.4	57.65 ± 1.2	57.8 ± 0.2
		5 mg/L	72.7 ± 0.5	76.47 ± 0.4	71.1 ± 0.5
		10 mg/L	94.5 ± 0.2	94.12 ± 0.2	88.5 ± 1.0
	Ridomil gold plus	2 g/L	63.3 ± 0.5	66.2 ± 0.5	70.7 ± 1.0

### Effect of AgNPs on pathogenic *Alternaria solani* mycelial morphology

**Fig. 7:**
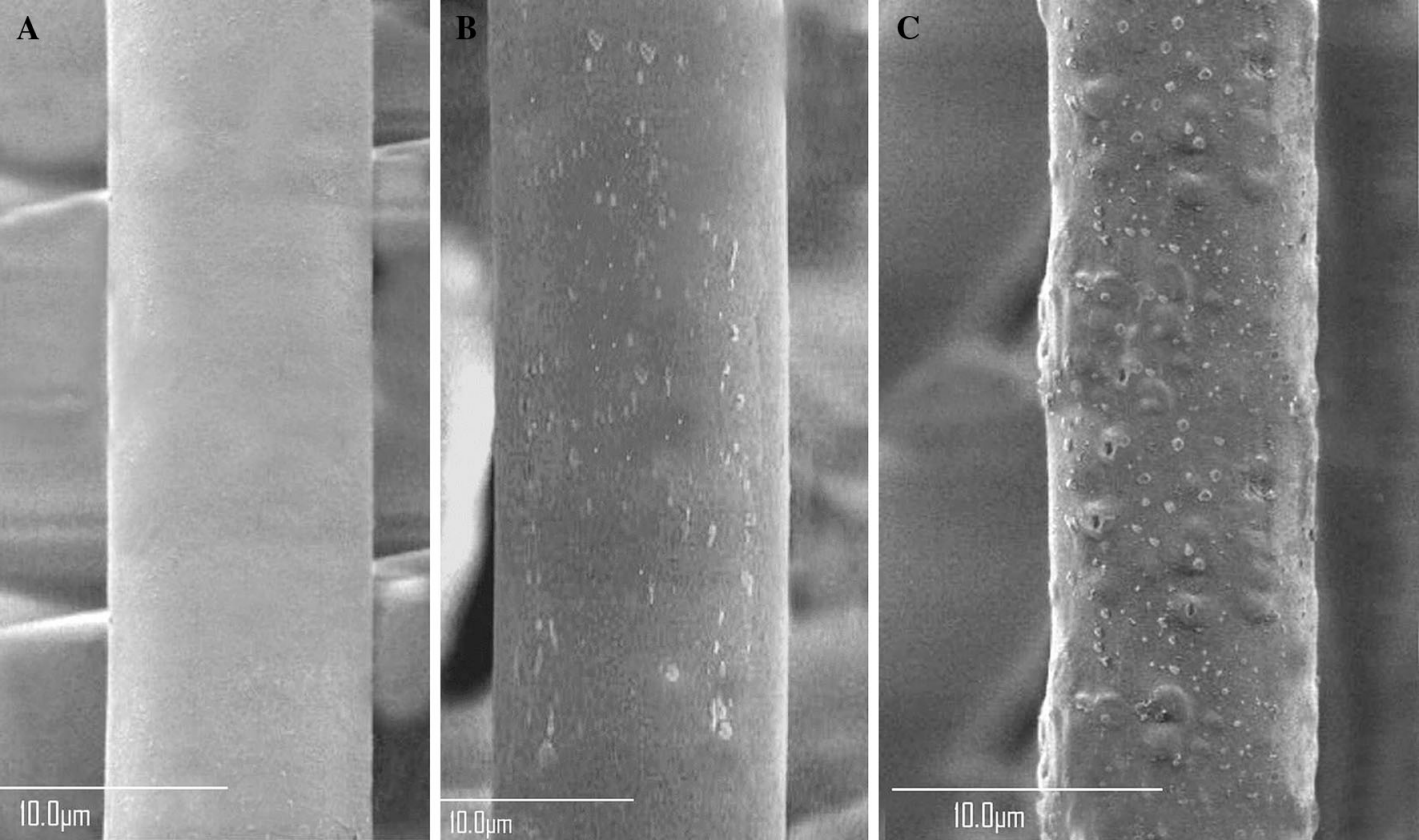
FE-SEM micrographs of pathogenic *Alternaria solani* F11 (KT721909) hyphae. **a** Fungal hyphae before treatment with AgNPs which showing regular and smooth surface. **b** and **c** Fungal hyphae after treatment with AgNPs. Pores and cavities were formed on the surface

The antifungal activity of the mycosynthesized AgNPs was studied using FE-SEM (Fig. 7). The FE-SEM micrographs of pathogenic *Alternaria solani* F11 (KT721909) hyphae before and after the treatment with AgNPs showed dense AgNPs around the mycelium cell wall and significant morphological changes in cell wall surface of the fungal pathogen (Fig. 7b, c), compared to the control displaying regular and smooth morphology (Fig. 7a). Pores and cavities were observed on the surface of treated fungal hyphae (Fig. 7c). The formation of pits may lead to creation of pores on the membranes. One possible cause is the reaction of AgNPs with phosphorous and sulfur containing materials inside and outside of cells. In addition, positive charge-containing AgNPs are believed to bind with negative charge-containing fungal membranes and disrupt cell walls and then destroying the membrane lipid bilayer, leading to induce the intracellular ion efflux resulting in cell death (Kanmani and Lim 2013).

**Fig. 8:**
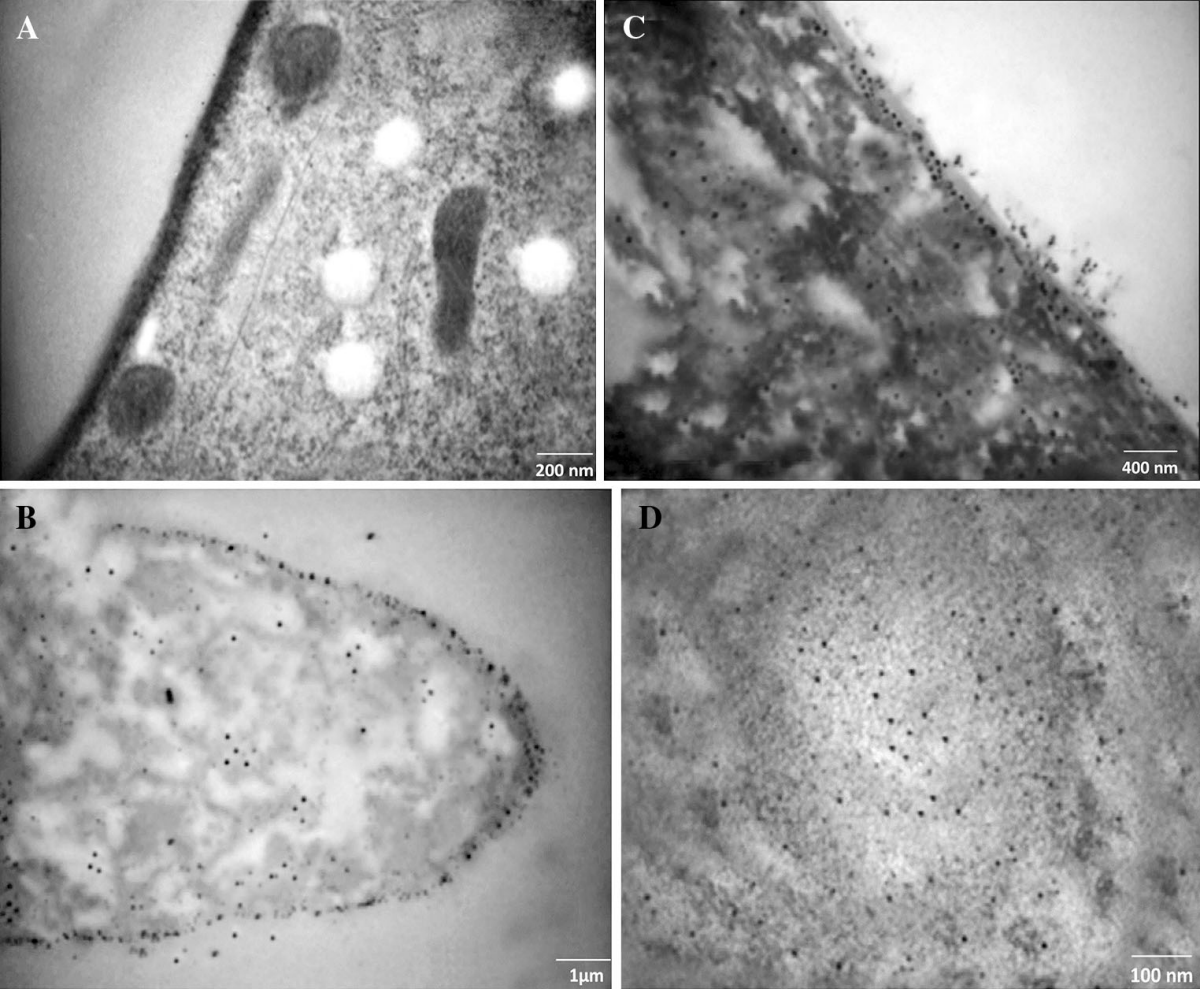
HR-TEM micrographs of pathogenic *Alternaria solani* F11 (KT721909) fungal cell. **a** Control sample of untreated fungi showing regular distribution and well distinguished cell components. **b, c** and **d** AgNPs treated *Alternaria solani*. **b** Numerous AgNPs accumulated on the outer region of the fungal cell wall. **c** Distribution the AgNPs in the cytoplasm and membrane. **d** Accumulation of AgNPs in the nucleus

The HR-TEM investigations indicated that the mycosynthesized AgNPs are able to pass through the fungal cell wall layers, distributing throughout the cell area and also interacting with the cell components (Fig. 8). In comparison to the control sample showed in Fig. 8a, numerous AgNPs were found accumulating on the outer region of the cell wall and then penetrate the fungal cell layers and distribute inside the cell area of treated fungi (Fig. 8b). AgNPs were also accumulating in cytoplasm and the cytoplasmic membrane and interacting with the cell components making major morphological changes (Fig. 8c). AgNPs accumulation was also observed in the cell nucleus (Fig. 8d), opening the possibility of interaction with DNA. These results are in accordance with the findings of Vahdati and Sadeghi 2013, showing the action of AgNPs on plasmid DNA in *Escherichia coli*. Moreover, these results backed to the fact that Ag itself has a greater affinity to sulfur and phosphorus containing biomolecules in the cell. Thus, sulfur containing proteins in the cell membrane, inside the cells and phosphorus containing elements like DNA are likely to be the preferred sites for binding of AgNPs (McDonnell and Russell 2001; Zhao et al. 2010).

### Effect of AgNPs on nucleic acid

**Fig. 9:**
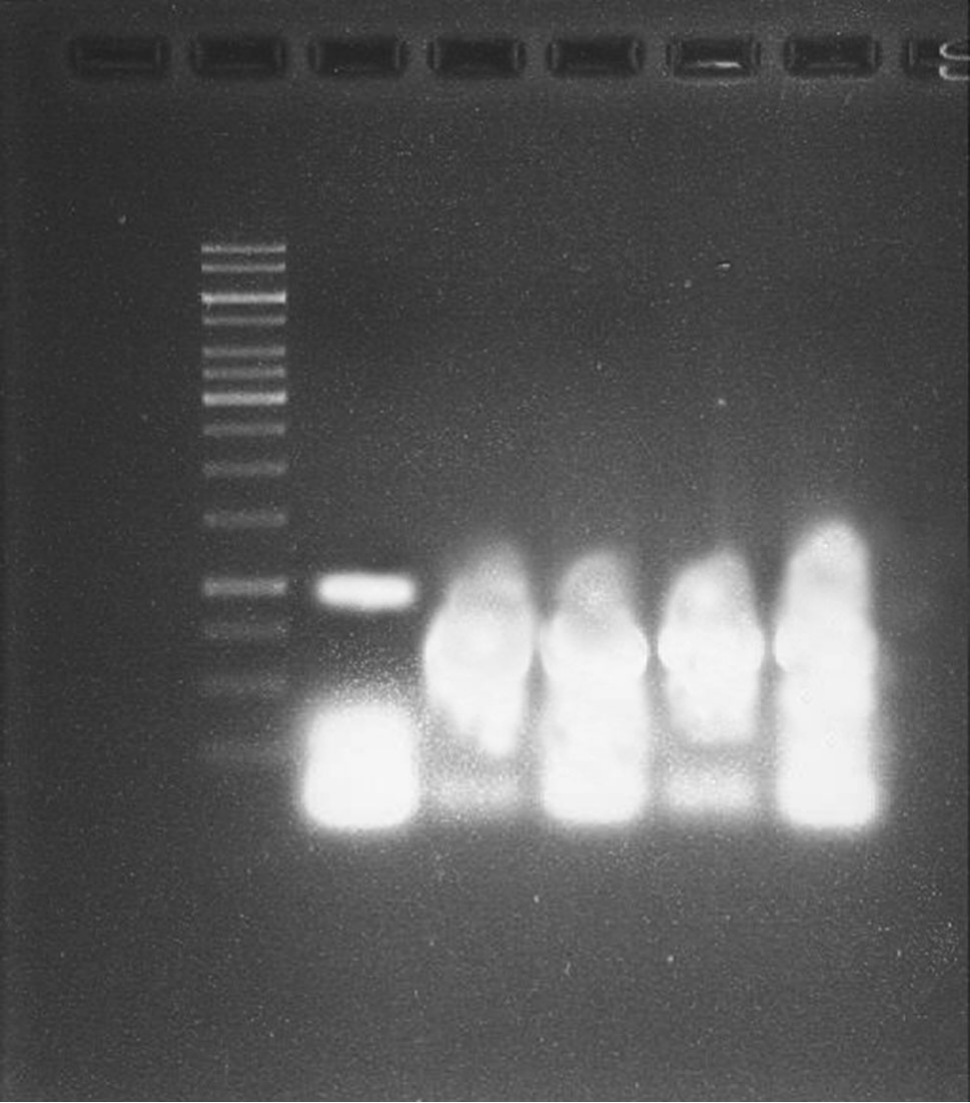
Gel electrophoresis analysis of AgNPs on DNA. After electrophoresis, the 1 % agarose gel was stained with ethidium bromide.* Lane 1*, DNA size marker;* lane 2*, control DNA non-treated with AgNPs;* lanes 3* to 6, DNA treated with AgNPs

To analyze the effect of mycosynthesized AgNPs on DNA, we set up a gel electrophoresis analysis. The results showed intact DNA bands with the untreated DNA (control), where no significant damage ocurred. In contrast, DNA treated with AgNPs (four replicates) showed substantial alteration in electrophoretic migration (Fig. 9). These results suggest that the biocidal effect of AgNPs may occur by direct chemical damage to DNA. So, AgNPs may act on the cell membrane of the pathogenic *Alternaria solani*, penetrating it, followed by damage and inhibition of DNA replication.

These results are in accordance with the findings by Vahdati and Sadeghi 2013, showing action of AgNPs on plasmid DNA of *E. coli*. Recent studied revealed the effect of AgNPs on *E. coli*, which could penetrate into the nucleic acid DNA of *E. coli* and exhibit antibacterial effect by causing DNA damage at low concentrations (Ramamurthy et al. 2013). Moreover, nanoparticles are also known to induce oxidative stress in microbes which will eventually lead to the killing of microbes. It has been previously reported that increased reactive oxygen species (ROS) production due to AgNPs damage membranes, forming free radicals with a powerful bactericidal action (Wu et al. 2014).

## Conclusions

A simple, fast, cost-effective, eco-friendly and stable method for mycogenic synthesis of AgNPs was successfully developed in the present work using non-pathogenic endophytic fungus *Alternaria solani* isolated from healthy tomato leaves. These mycosynthesized AgNPs are homogenous and stable for longer duration as determined by UV–vis spectroscopy, HR-TEM, XRD and FT-IR analysis, indicating that entophytic fungal extract can be used as reducing/capping agents to prevent aggregation and increase the stability of AgNPs. Interestingly, the mycosynthesized AgNPs at (5 and 10 ppm) exhibited a more effective activity in inhibiting the mycelia growth of pathogenic strains of *Alternaria solani*, the causal agent of tomato early blight disease as compared to the recommended chemical fungicide used (Ridomil gold plus 2gL^−1^). Damage of fungal hyphae structure was observed through electron microscopy in hyphae treated with the mycosynthesized AgNPs. The mechanism of AgNPs antifungal activity may be related to damaging the fungus membrane lipid bilayer, leading to intracellular ion efflux resulting in cell death. Also, accumulation of AgNPs in the cell nuclei and interaction with DNA may lead to cell death. All these findings give scope for the possible development of formulations containing AgNPs as effective nano bio-fungicides, alternatives to chemical compounds. Also, further, developed and modified porous nanoparticles could be exploited for delivery of effective fungicides. Thus, exhaustive experimental trials on plants are needed to prove the field applicability of the synthesized nanoparticles in plant disease management.
